# A functional variant in the 3ˈ-UTR of *VEGF* predicts the 90-day outcome of ischemic stroke in Chinese patients

**DOI:** 10.1371/journal.pone.0172709

**Published:** 2017-02-24

**Authors:** Jing Zhao, Yun Bai, Lei Jin, Yingfeng Weng, Yujie Wang, Hui Wu, Xia Li, Ying Huang, Shengyue Wang

**Affiliations:** 1 Department of Neurology, Minhang district central hospital, Shanghai, China; 2 Shanghai-MOST Key Laboratory of Health and Disease Genomics, Chinese National Human Genome Center at Shanghai, Shanghai, China; 3 School of Life Sciences, Fudan University, Shanghai, China; 4 Luoxin Biotechnology Company at Shanghai, Pudong New Area, Shanghai, China; Huazhong University of Science and Technology, CHINA

## Abstract

Vascular endothelial growth factor (VEGF) plays critical roles in angiogenesis and vasculogenesis, which are associated with post-stroke functional recovery. However, the effects of the *VEGFA* polymorphisms on the outcome of ischemic stroke (IS) have been rarely reported. We therefore investigated the associations of +936*C*/*T* variant (rs3025039) with the susceptibilities and the 90-day outcomes from 494 IS patients and 337 healthy controls in Chinese population through the establishment of logistic multivariate regression model. Stroke severity at admission and outcome of 90 days were respectively assessed according to the National Institutes of Health Stroke Scale and the modified Rankin Scale. The analysis showed that there were no significant associations of the rs3025039 genotypes with the susceptibility (*P* = 0.229) and the severity (*P* = 0.734). However, when we divided the 308 IS patients into two groups according to the different outcomes, we found that the rs3025039 *TC*+*TT* genotype significantly increased the risk of poor recovery [adjusted odds ratio (OR), 1.99; 95% confidence interval (CI), 1.18–3.37]. Interestingly, we observed another 3ˈUTR variant, +1451*C*/*T* (rs3025040), exhibited strong linkage disequilibrium (r^2^ = 1.0) with +936*C*/*T* and was located in a predicted microRNA-binding site. The rs3025040 *T* allele significantly decreased the luciferase activities in four cell lines, which indicated a potential disruption of the miRNA-mRNA interaction that would result in lower VEGF expression levels. Our data suggested that the +936*C*/*T* variants significantly increased the risk of poorer stroke outcome by affecting the bindings of miR-199a and miR-199b to *VEGF* mRNA at the rs30250340 polymorphic site.

## Introduction

Stroke is one of the principal reasons leading morbidity and mortality worldwide [1]. In China, the prevalence of stroke is 2.5 million per year, and the annual stroke mortality rate is approximately 0.157%, which exceeds that of heart disease [2]. It is estimated that more than 80% of stroke cases originate from ischemic strokes (IS) [3], which is a complex disorder caused by multiple genetic and environmental factors [4]. Two genome-wide association studies (GWAS) have been performed to identify the susceptibility loci for IS risk [5,6]. Some chromosome loci, such as 6p21.1, 9p21 and 7p21.1, have been demonstrated to affect one or more subtypes of the stroke. But what is more interesting from clinic is the similar locations or extents of damage in IS patients with different functional outcomes. This observation indicates that genetics might contribute to the differences in the rates of stroke recovery [7]. However, there are still very few studies of the genetic factors that contribute to stroke recovery.

Vascular endothelial growth factor (VEGF), also known as vascular permeability factor, is one of the key initiators and regulators of angiogenesis and vascularization [8]. In response to ischemic injury, VEGF can induce contralesional corticobulbar plasticity and functional neurological recovery [9]. Several studies have demonstrated temporal or spatial correlations between the up-regulations of VEGF or VEGF receptors and angiogenesis following the occlusion of the middle cerebral artery in rats [10–12]. Additionally, the administration of VEGF during the early post-ischemic phase stimulates both angiogenesis and neurogenesis and leads to improved functional recovery following stroke. For example, intravenous VEGF increases microvessel density in the cortical ischemia penumbra [13], and intracerebroventricular VEGF enhances the delayed survival of newborn neurons in the dentate gyrus and subventricular zone [14]. Moreover, the stimulation of VEGF expression has been implicated in the neurogenesis-promoting which has effects of therapeutic agents, such as statins [15] and angiotensin II receptor blockers [16].

Given the importance of the roles of VEGF in the brain repair following stroke, we hypothesized that genetic variants may interrupt VEGF expression and contribute to poor stroke recovery. Although a previous study confirmed that genetic polymorphisms in the *VEGFA* gene may be associated with the risk of IS [17], the effects of *VEGFA* genetic variants on 90-day outcomes of IS have not been reported.

## Materials and methods

### Ethics statement

All procedures performed in studies involving human participants were in accordance with the ethical standards of the institutional and/or national research committee. The ethics committee of Minhang District Central Hospital approved this study. Each participant or their legal representatives consent to participate by signing an informed consent form. This article does not contain any studies with animals performed by any of the authors. Informed consent was obtained from all individual participants included in the study.

### Study population

The subject recruitment has been described elsewhere [18]. Briefly, the study design included two components: a case-control study with 494 IS patients and 337 health controls, and a prospective 90-day follow-up of the functional disabilities of 308 IS patients. All patients who were diagnosed between 2008 and 2012 according to the World Health Organization’s definition of stroke [19] in Shanghai Minhang District Central Hospital were recruited. The diagnoses were based on clinical manifestations and the results of cranial computed tomography (CT) or magnetic resonance image (MRI) performed within 2 days of onset. The exclusion criteria included hemorrhagic stroke, transient ischemic attack and the inability to undergo cranial CT/MRI imaging. In addition to the neurological, hypertension, diabetes mellitus (DM), and hypercholesterolemia histories, the following vascular risk factors were also recorded for each individual: blood pressure, body mass index (BMI), cigarette smoking, alcohol consumption, and serum glucose and total cholesterol levels. All of the information was obtained from medical records, the current use of corresponding medications, or clinical examination results after admission. Age- and sex-matched healthy control subjects attending physical check-ups who did not exhibit IS signals on cranial CT/MRI and had no history of stroke or myocardial infarction were recruited. All subjects were genetically unrelated ethnic Han Chinese individuals.

All IS patients were classified based on the Trial of Org 10172 in Acute Stroke Treatment (TOAST) definitions [20]. The severity assessments were performed on the first day (i.e., the day of admission) based on the National Institutes of Health Stroke Severity Scale (NIHSS)[21]. Mild stroke was defined as a NIHSS of 6 or lower and severe stroke as a NIHSS of 7 or higher because better prognoses have frequently been observed in patients with scores ≤ 6. Post-stroke disability was assessed at 3 months after stroke using the Modified Rankin Scale (mRS) [22]. A good outcome was defined by mRS ≤ 1, and a poor outcome was defined by mRS ≥ 2. The outcomes were measured at 90 days post-stroke because the majority of IS patients exhibit maximum rehabilitation of neural repair and the greatest genetic influence at this time.

### DNA isolation and *VEGFA* +*936C* > *T* genotyping

Genomic DNA was extracted from 200μl of EDTA-treated blood using a Blood Genome DNA Extraction Kit (TaKaRa Biotechnology, Dalian) according to the manufacturer’s instructions and stored at -20°C as needed. Genotyping was performed with a PCR/ligase detection reaction assay. The PCR primers and probes were designed using the web-based version of the Primer 3.0 program (http://frodo.wi.mit.edu/primer3/). The rs3029039 SNP was amplified with the following pair of primers: forward, 5ˈ-ACACCATCACCATCGACAGA -3ˈ; and reverse, 5ˈ-GGCTCGGTGATTTAGCA-3ˈ. PCR amplification was performed in a 20 μl reaction volume containing 1 μl of genomic DNA, 2 μl of 10×PCR amplification buffer, 2 μl of 2.0 mM dNTP mixture, 0.4 μl of each primer (10 pmol/μl), and 0.3 μl of Taq DNA polymerase (5 U/μl, TaKaRa Biotechnology, Dalian). The PCR reaction was initiated at 95°C for 15 min followed by 35 cycles of denaturation at 94°C for 30 s, annealing at 56°C for 30s, and extension at 72°C for 1 min, followed by a final extension at 72°C for 10 min. The purified PCR products were then sequenced on an ABI 3730 XL sequencer (Applied Biosystems, USA).

### Expression and luciferase reporter constructs

Based on the GenBank *VEGFA* mRNA sequence (accession number NM_001025366), the 3ˈUTR of wild-type *VEGFA* with the +936*C* and +1451*C* was amplified from the genomic DNA using the following primers: forward, 5ˈ-GTAGACGCGTGGGAACCAGATCTCTCACCA-3ˈ; and reverse, 5ˈ-TCCAAAGCTTGGGCAGAGCTGAGTGTTAGC-3ˈ. The PCR product was cloned into the pMIR-REPORT luciferase miRNA expression reporter vector (Applied Biosystems, USA) and sequenced to confirm the resulting plasmid pMIR-*C*/*C* (wild-type).

After WT cloning, a Muta-direct^™^ Site-Directed Mutagenesis Kit (Beijing SBS Genetech, China) was used to generate the +936*C*>*T* and +1451*C*>*T* mutation in the 3’UTR of the *VEGFA* gene via PCR using the WT *VEGFA* construct pMIR-*C*/*C* as the template. Primers containing the mutations +936*C*>*T* and +1451*C*>*T* were designed and used for site-directed mutagenesis as follows: +936*C*>*T* forward: 5ˈ-GGCGGGTGACCCAGCA**T**GGTCCCTCTTGGAATT-3ˈ and +936*C*>*T* reverse: 5ˈ-AATTCCAAGAGGGACC**A**TGCTGGGTCACCCGCC-3ˈ; and +1451*C*>*T* forward: 5ˈ-ACAGGGATGAGGACAC**T**GGCTCTGACCAGGAGT-3ˈ and +1451*C*>*T* reverse: 5ˈ-ACTCCTGGTCAGAGCC**A**GTGTCCTCATCCCTGT-3ˈ (the mutations are underlined). The construct was designated pMIR-*T*/*T* (mutant type).

The microRNA (miRNA) expression reporter plasmids were constructed in a similar manner. Precursors of miR-199a and miR-199b were amplified by PCR using the following primers: miR-199a forward: 5ˈ-TCCAAAGCTTGACCCCCAAAGAGTCAGACA-3ˈ and miR-199a reverse: 5ˈ-CTAGTCTAGACTTTCCCCAGTGCCTCTTCT-3ˈ; and miR-199b forward: 5ˈ-TCCAAAGCTTCACGTCAAAGGAGGCAGAAG-3ˈ and miR-199b reverse: 5ˈ-CTAGTCTAGAGAGTGTCAAGGTGCGTGTGT-3ˈ. The products were cloned into pcDNA3.1(+) plasmids with HindIII and Xba I digestion and sequenced as pcDNA-miR-199a and pcDNA-miR-199b.

### Cell culture, transfection and luciferase reporter assay

Human HEK293T cells (human embryonic kidney 293 cell line), A549 cells (human lung adenocarcinoma cell line), 16HBE cells (human bronchial cell line) and ECV-304 cells (human vein endothelial cell line) were respectively maintained in DMEM, F-12K and DMEM media at 37°C in a humidified air with 5% CO_2_. All of these media were supplemented with 10% fetal bovine serum, penicillin (100 U/ml) and streptomycin (100 mg/ml).

One day before transfection, the growing cells were seeded in a 24-well plate at a density of 1×10^6^ cells/ml. The next day, cells were cotransfected with firefly luciferase reporter vector (pMIR-*C*/*C* or pMIR-*T*/*T*), microRNA expression reporter plasmids (pcDNA-miR-199a, pcDNA-miR-199b or the negative control pcDNA3.1) and pRL-SV40 plasmids (Promega, Madison); the latter plasmids were expressed Renilla luciferase to enable the monitoring of transfection efficiency. For each well, 150ng luciferase plasmid, 300ng miRNA expression reporter plasmids and 50ng Renilla plasmid were transfected using 1 μl of Lipofectamine 2000 (Invitrogen) according the manufacturer’s protocol. After 48 h, the firefly and Renilla luciferase activities were measured in triplicate using the Dual-Luciferase Reporter Assay system (Promega, Madison) with a MicroLumatPlus LB 96V (Berthold Technologies, Germany).

### Statistical analysis

The demographic data, clinical characteristics, NIHSS score on admission, and mRS outcome scores at 90 days were compared using t-tests or χ2 tests as appropriate. The Hardy-Weinberg equilibrium of the alleles was assessed with a goodness-of -fit χ^2^ test with one degree of freedom to compare the observed genotypes frequencies with the expected ones among the controls. Multiple logistic regression analyses were used to determine the effects of the genetic variables on the IS outcomes after adjusting for significant non-genetic variables, including age, sex, blood pressure, cigarette smoking, serum glucose, total cholesterol levels and severity at admission. Logistic regression models were estimated by computing the odds ratios (ORs) and 95% confidence intervals (CIs). All statistical tests were performed using SPSS19.0 software (SPSS, Inc., USA).

## Results

### Clinical and laboratory characteristics

The general characteristics of the patients and controls are provided in Table 1. There were 300 males and 194 females in the case group, and the mean age of the IS group was 69.75±11.32 years (range from 40 to 85). The control group included 185 males and 152 females, and the mean age was 68.96±9.99 years (range from 40 to 85). There were no statistically significant differences in age (*P* = 0.303) or gender (*P* = 0.109) between the case and control groups. The frequencies of the traditional cerebrovascular risk factors in the IS group were as follows: hypertension (75.7%), DM (34.0%), hypercholesterolemia (33.2%), and smoking (40.9%). These frequencies were significantly higher than those of control group (*P* < 0.01). Next, all IS patients were classified into four subtypes according to the TOAST criteria. Two hundred (40.5%) were classified as large artery atherosclerosis (LAA), 165 (33.4%) as small artery occlusion (SAO), 82 (16.6%) as cardioembolism, and 47 as the other subtypes due to the small sample sizes. No significant differences were noted between the ages or genders of each of the subtypes groups and the control group. However, hypertension and DM were still risk factors in the each of the subtypes compared with the controls.

**Table 1:** General characteristics of IS cases and controls.

Characteristic	Control	Ischemic stroke		TOAST Subtypes					
		Total	*P* value	LAA	*P* value	SAO	*P* value	Cardioembolism	*P* value
Number	337	494		200		165		82	
Age (years)*	68.96±9.99	69.75±11.32	0.303	70.03±10.721	0.067	68.39±10.78	0.362	70.99±13.09	0.124
Gender, M/F ^†^	185/152	300/194	0.109	129/71	0.098	100/65	0.162	42/40	0.319
Hypertension, n (%)^†^	205 (60.8%)	374 (75.7%)	<0.001	152 (76.0%)	<0.001	130 (79.8%)	<0.001	57 (69.5%)	0.145
Diabetes mellitus, n (%)^†^	56 (16.6%)	168 (34.0%)	<0.001	65 (32.5%)	<0.001	54 (33.1%)	<0.001	30 (36.6%)	<0.001
Hypercholesterolemia, n (%)^†^	80 (23.7%)	164 (33.2%)	0.003	56 (28.0%)	0.258	51 (30.9%)	<0.001	22 (26.8%)	0.019
Smoking, n (%)^†^	91 (27.0%)	202 (40.9%)	<0.001	108 (54.0%)	<0.001	79 (49.4%)	0.309	37 (45.7%)	0.849

TOAST indicates the Trial of Org 10172 in Acute Stroke Treatment; LAA, large artery atherosclerosis; SAO, small artery occlusion.

* t-tests for the differences between ischemic stroke patients and control subjects

^†^ Chi-square test for the differences in the distribution frequency between ischemic stroke patients and control subjects

### Association of the *VEGFA* gene +936 (rs3025039) genotype with the susceptibility of IS

Genetic analysis of the *VEGFA* rs3029039 polymorphism was performed for all 494 patients and 337 controls. The genotypes of 17 controls were not determined due to low qualities or quantities template DNA. Therefore, a total of 320 controls were genotyped in this study. The genotype and allele frequency distributions of the +936 *C*>*T* substitution in the IS patients and control subjects are presented in Table 2. The polymorphism frequencies in the control subjects were consistent with the Hardy-Weinberg equilibrium expectation (*P* = 0.187). We calculated the adjusted OR using multiple logistic regression analysis with adjustments for the traditional risk factors, including age, sex, hypertension, DM, hypercholesterolemia, and smoking. The results suggested that subjects with the +936 *TT* genotype had a higher risk of IS than the subjects carrying the *CC* genotype (adjusted OR = 1.50; 95% CI, 0.95–2.36; *P* = 0.082). There were no significant associations of the rs3025039 *CT+TT* genotype with the susceptibility of IS or its subgroups.

**Table 2:** Genotype frequency of *VEGFA* polymorphisms between IS patients and control subjects.

VEGF genotypes	Controls	Cases	TOAST Subtypes			OR (95%CI) †			
			LAA	SAO	Cardioembolism	All cases	LAA	SAO	Cardioembolism
Number	320	494	200	165	82				
rs3025039									
* CC*	104 (32.5%)	168 (34.0%)	71 (35.5%)	66 (40.0%)	22 (26.8%)	1.00 (reference)	1.00 (reference)	1.00 (reference)	1.00 (reference)
* CT*	176 (55.0%)	232 (47.0%)	94 (47.0%)	66 (40.0%)	44 (53.7%)	0.83 (0.61–1.15)	0.87 (0.59–1.28)	0.62 (0.40–0.95)	1.28 (0.71–2.30)
* TT*	40 (12.5%)	94 (19.0%)	35 (17.5%)	33 (20.0%)	16 (19.5%)	1.50 (0.95–2.36)	1.17 (0.67–2.07)	1.49 (0.83–2.66)	2.45 (1.11–5.41)*
*CC* vs *CT*+*TT*						0.95 (0.70–1.30)	0.83 (0.57–1.20)	0.94 (0.64–1.37)	1.48 (0.84–2.60)

TOAST indicates the Trial of Org 10172 in Acute Stroke Treatment; LAA, large artery atherosclerosis; SAO, small artery occlusion; OR, adjusted odds ratio; 95%CI, 95% confidence interval.

^†^ OR based on the risk factors, including age, sex, blood pressure, cigarette smoking, serum glucose, total cholesterol levels and severity at admission.

* *P* < 0.05

### Association of the *VEGFA* gene +936 (rs3025039) genotype with the severity and outcome of IS

All the patients were divided into two subgroups according to IS severity (n = 382, NIHSS score ≤ 6 *vs*. n = 112, NIHSS score > 6). We did not find any association of the *CT* genotype (adjusted OR = 0.79; 95% CI 0.50–1.25; *P* = 0.317), *TT* genotype (adjusted OR = 0.72; 95% CI 0.41–1.27; *P* = 0.256) or *T* allele (OR = 0.80; 95% CI 0.59–1.09; *P* = 0.167) with IS severity (Table 3). We then stratified all patients according to the different outcomes. Of the 494 stroke patients, 308 (62.3%) were assessed at 90 days after the acute event. Eight patients (1.6%) died, and 11 patients (2.2%) had a recurrent IS. These 308 patients were classified into good recovery (n = 193) and poor recovery groups (n = 115) based on the mRS assessments. As presented in Table 4, we found a significant difference between these two groups in the allele frequencies (OR = 1.50; 95% CI 1.08–2.09; *P* = 0.015). Following further adjustments for some non-genetic variables, including age, sex, hypertension, DM, hypercholesterolemia, smoking and IS severity, we found that the patients with the *CT* or *TT* genotypes exhibited poorer outcomes than the *CC* carriers (adjusted OR = 1.98; 95%CI 1.13–3.48; *P* = 0.017 for the *CT* genotype and adjusted OR = 2.16; 95%CI 1.10–4.22; *P* = 0.024 for the *TT* genotype). The *CT*+*TT* genotypes significantly increased the risk of a bad outcome compared with the *CC* genotype (OR = 1.99; 95%CI 1.18–3.37; *P* = 0.011).

**Table 3:** Genotype and allele frequencies of rs3025039 of *VEGFA* gene in IS patients with different severity at 1 day.

VEGF genotypes	IS patients (n = 308)		OR(95%CI)	*P* value
	NIHSS ≤ 6 (n = 382)	NIHSS > 6 (n = 112)		
rs3025039				
* CC*	128 (33.5%)	40 (35.7%)	1.00 (reference)	
* CT*	174 (45.5%)	58 (51.8%)	0.79 (0.50–1.25) ^†^	0.317
* TT*	80 (20.9%)	14 (12.5%)	0.72 (0.41–1.27) ^†^	0.256
*CT*+*TT*	254 (64.5%)	72 (64.3%)	0.91(0.58–1.41)	0.734
Allele				
* C*	430 (56.3%)	138 (61.6%)	1.00 (reference)	
* T*	334 (43.7%)	86 (38.4%)	0.80 (0.59–1.09)	0.167

NIHSS indicates the National Institutes of Health Stroke Severity Scale assessed at one day; OR, odds ratio; 95%CI, 95% confidence interval.

^†^ OR were adjusted by the risk factors, including age, sex, blood pressure, cigarette smoking, serum glucose, total cholesterol levels and severity at admission.

**Table 4:** Genotype and allele frequencies of rs3025039 of *VEGFA* gene in IS patients with different outcome at 90 day.

VEGF genotypes	IS patients (n = 308)		OR(95%CI)	*P* value
	mRS ≤ 1 (n = 193)	mRS > 1 (n = 115)		
rs3025039				
* CC*	71 (36.8%)	26 (22.6%)	1.00 (reference)	
* CT*	85 (44.0%)	60 (52.2%)	1.98 (1.13–3.48) ^†^	0.017*
* TT*	37 (19.2%)	29 (25.2%)	2.16 (1.10–4.22) ^†^	0.024*
*CT*+*TT*	122 (63.2%)	89 (77.4%)	1.99 (1.18–3.37)	0.011
Allele				
* C*	227 (58.8%)	112 (48.7%)	1.00 (reference)	
* T*	159 (41.2%)	118 (51.3%)	1.50 (1.08–2.09)	0.015*

mRS indicates the Modified Rankin Scale assessed at 90 day; OR, odds ratio; 95%CI, 95% confidence interval.

^†^ OR were adjusted by the risk factors, including age, sex, blood pressure, cigarette smoking, serum glucose, and total cholesterol levels.

* *P* < 0.05.

### Linkage disequilibrium analysis across populations

**Fig 1:**
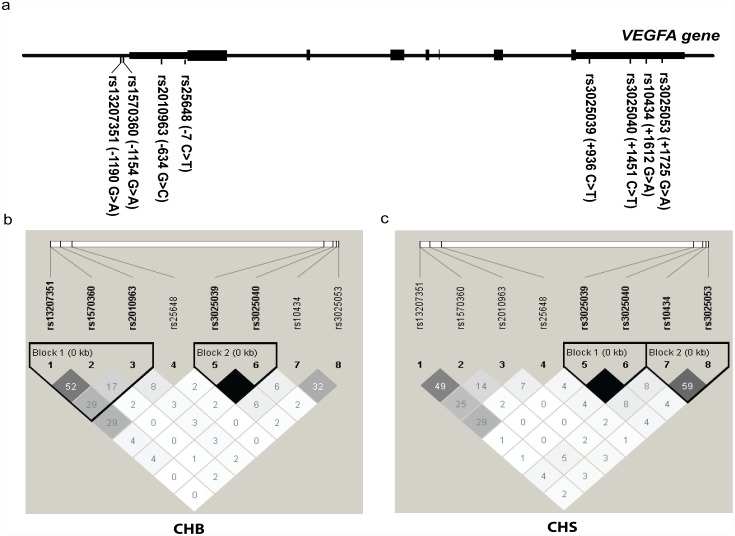
Gene map, polymorphisms and linkage disequilibrium (LD) coefficients. (a) A gene map and the polymorphisms of the *VEGFA* gene on chromosome 6p21. The reference genomic sequence was NM_001025366. (b, c) The LD structure of eight polymorphisms within the *VEGFA* region in the north (CHB, n = 97) and south (CHS, n = 100) Chinese populations. The LD plot was generated using Haploview v4.2 (Broad Institute, Cambridge, MA, USA). The degree of pairwise LD (r^2^) is also shown in each block. rs3025039 and rs3025040 form a haplotype block with a complete LD value (r^2^ = 1.0).

To better understand the associations above, we extracted Chinese data from the 1000 genomes browser (http://browser.1000genomes.org/index.html). Eight potentially functional *VEGFA* polymorphisms with high heterogeneities in minimal allele frequencies (MAF > 0.2) were selected (Fig 1a). Among these polymorphisms, two were located in the promoter region (rs13207351 and rs1570360), two in the 5ˈUTR (rs2010963 and rs25848) and four in the 3ˈUTR (rs3025039, rs3025040, rs10434, and rs3025053). We performed linkage disequilibrium (LD) analyses to test for the presence of bins in the region encompassing these SNP. As illustrated in the LD plots in Fig 1b and 1c, we found that +1451 *C* > *T* (rs3025040) presented a perfect LD value (r^2^ = 1.0) with +936 *C* > *T* (rs3025039) in the north (CHB, n = 97) and south (CHS, n = 100) Chinese populations. Additionally, through ABI 3730XL DNA sequencing, we also noticed a strong LD (r^2^ = 1.0) relationship between +936 *C* > *T* and +1451 *C* > *T* in 36 patients in the IS group (S1 Table), which suggests that these patients may exhibit the epidemiological results in terms of genetic susceptibility and outcome of IS.

### Bioinformatics analysis results

**Fig 2:**
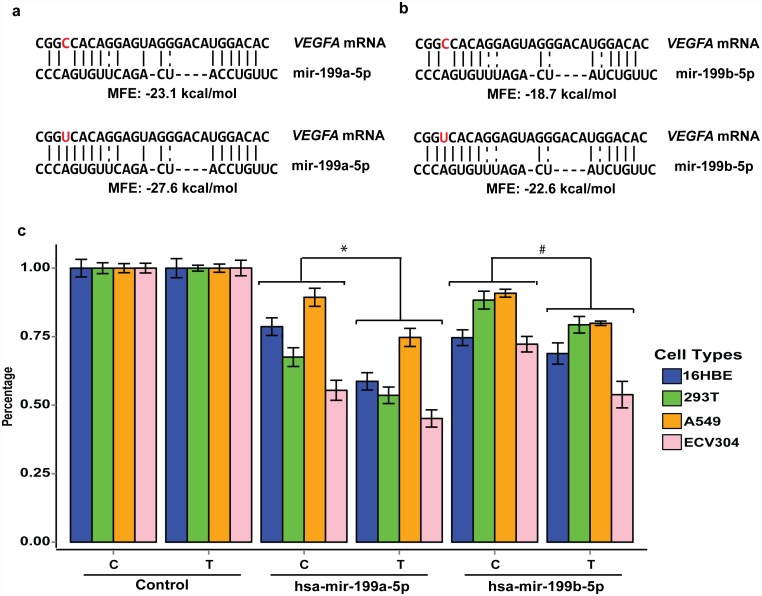
SNP +1451 *C*>*T* affects *VEGFA* expression by interfering with miR-199a-5p and miR-199b-5p function. (a, b) miR-199a-5p and miR-199b-5p directly target the *VEGFA* gene 3ˈUTR region and +1451*C* > *T* (rs3025040) is located in this binding site and may interfere with miRNA-mRNA interactions due to the base substitution. MFE: minimal free energy. (c) In vitro luciferase reporter assays for +1451*C* > *T* in the 16HBE, 293T, A549 and HEV304 cell lines. Each transfection was performed with pRL-SV40 plasmids as normalizing controls. The data are presented the mean fold increases ± SDs relative to the samples co-transfected with the pcDNA3.1empty vector (control) from three independent transfection experiments, each of which was performed in triplicate. Pairwise t test was used to evaluate the differences between the wild-type and mutant-type. * hsa-miR-199a expression plasmid with *VEGFA* wild-type 3’UTR luciferase reporter plasmids (pMIR-*C*/*C*) or mutant plasmids (pMIR-*T*/*T*) (*P* = 0.062, *P* = 0.002, *P* = 0.083, and *P* = 0.107 for 16HBE, 293T, A549, and ECV304, respectively. *P* < 0.001 for combined all four cell lines). ^#^ hsa-miR-199b expression plasmid with *VEGFA* pMIR-*C*/*C* plasmid or pMIR-*T*/*T* plasmids (*P* = 0.153, *P* = 0.145, *P* < 0.001, and *P* = 0.130 for 16HBE, 293T, A549, and ECV304, respectively. *P* < 0.001 for combined all four cell lines).

Since both +936 *C* > *T* and +1451 *C* > *T* were located in the 3ˈUTR of the *VEGFA* gene, we hypothesized that these genetic variants may interrupt miRNA-mRNA interactions and affect VEGF expression. Therefore, we searched for miRNAs with bindings that could be affected by the +936 *C* > *T* or +1451 *C* > *T* base substitution using MirSNP database [23]. We identified miR-591 and miR-199 family as containing seed sequences that respectively corresponded to the complementary sequences surrounding +936 *C* > *T* and +1451 *C* > *T*. Further using RNAhybid [24], which is an online tool for identifying the minimum free energy (MFE) hybridization of a long and a short RNA, we noticed that the +1451 *T* allele exhibited significantly lower MFE values than the C allele for both miR-199a (-27.6 vs -23.1 kcal/mol) and miR-199b (-20.1 vs -15.6 kcal/mol) (Fig 2a and 2b). Additionally, we also found that the allele change from +936 C -> T is only slightly increased the binding of VEGF 3’UTR with miR-591 (-22.1 vs -23.3 kcal/mol). This alter is significantly smaller than miR-199a or miR-199b (S1 Fig). These results suggested that the +1451 *T* allele may change the conformation of the secondary structure of *VEGFA* and may increase the binding affinities between the *VEGFA* mRNA and the miRNAs (hsa-miR-199a, hsa-miR-199b or both) compared with the +1451 *C* allele. Therefore, we only evaluated the binding of miR-199a and miR-199b with rs3025040 in the present study.

### hsa-miR-199a and hsa-miR-199b down-regulated the wild and mutated *VEGFA* 3’UTR luciferase activities

To further confirm *VEGFA* as a putative target of hsa-miR-199a or hsa-miR-199b and that the +1451 *C* > *T* substitution may affect hsa-miR-199a or hsa-miR-199b targeting, we constructed two luciferase reporter vectors with wild-type homozygous (wil: pMIR-+936*C*/+1451*C*, pMIR-*C*/*C*) and mutated homozygous (mut: pMIR-+936*T*/+1451*T*, pMIR-*T*/*T*) versions of the *VEGFA* gene and two expression plasmids, i.e., pcDNA3.1-miR199a and pcDNA3.1-miR199b. When these plasmids were co-transfected into 16HBE, A549, 293T and ECV304 cells, the relative luciferase activities of the *VEGFA* mutated luciferase reporter groups were suppressed by an average of 22.6% (hsa-miR-199a) and 14.4% (hsa-miR-199b) in all four types of cell lines, compared with the results observed following transfection with the wild-type (Fig 2c and S2 Table). This difference is more obvious as we combined all cell lines results (*P* < 0.001). These findings suggested that mut led to a lower VEGF expression level than wil. Moreover, compared with co-transfection with the pcDNA3.1empty vector, the relative luciferase activities were significantly decreased in the cells that were co-transfected with pcDNA3.1-miR199a or -miR199b. In contrast, no changes in relative luciferase activity were observed in the cells that were transfected with or without the pcDNA3.1 empty vector groups of wil and mut.

## Discussion

IS is a complex disease caused by a combination of multiple risk factors. Some previous findings suggested that genetic polymorphisms might play an important role in the risk for IS [6,25–27]. As a gene that putatively contributes to IS, VEGF has been highlighted in many in *vivo* and in *vitro* studies. The administration of recombinant human VEGF to the ischemic brain has been proven to promote angiogenesis and thereby improving functional neurological outcomes [13,14]. Several variants in the *VEGFA* promoter and exons have been reported to predispose carriers to the development of IS [17]. A recent meta-analysis also demonstrated that +936C>T may be a risk factor for stroke among Asians [28], even though inconsistent result was observed in a Chinese population [29]. In the present study, we also evaluated the distribution of the *VEGFA* +936 *C* > *T* polymorphism in 494 IS patients and 337 healthy control. There were no significant associations of the +936 *C* > *T* genotypes with the susceptibility. This result is inconsistent with what has been shown in some previous studies [28], which may be due to the selection bias of IS patients or some potential confounding factors that influenced the distribution of the genotype. Therefore, we further assessed the severity and the 3 month outcomes of IS in relation to the polymorphisms and found that the subjects with the *TT* genotype of rs3029039 exhibited an increased risk of poor outcome at 90 days after IS onset. These results further support previous ideas that VEGF is an important regulatory gene for the recovery from IS and that the +936 *C* > *T* substitution may be a functional polymorphism in the 3ˈUTR that may affect the outcomes of IS patients.

The *VEGFA* +936 *C* > *T* substitution is an important functional polymorphism that has been proven to alter susceptibility to various diseases that are linked to altered angiogenesis, including, endometriosis [30], cancer [31,32], and vascular and periaortic diseases [33,34]. A marked heritability of VEGF serum levels according to the +936 *C* > *T* genotype has been reported in a healthy Caucasian population [35]. Similar results were observed in another previous study [32] which suggested that the variant may affect VEGF levels through some specific transcriptional or post-transcriptional regulation. There are two interpretations of this phenomenon. First, the +936 *C* > *T* polymorphism, which is located in the 3ˈUTR, leads to the loss of a potential binding site for AP-4 [32,36]; Second, this variant may interfere with the binding of hypoxia-induced protein to the 3ˈUTR of *VEGFA* mRNA, which would result in a significantly decreased half-life of the mRNA [37].

In the present study, we hypothesized a different potential mechanism as an explanation. Because the 3ˈ-UTR contains regulatory sequences that are sensitive to regulatory proteins and miRNAs, mutations or polymorphisms in the miRNA binding site could result in alterations in the accessibility of miRNA for the binding of the target gene and thereby influence the risks for common diseases [38]. Several previous studies have identified associations of certain variants in miRNA binding site with complex trait diseases such as DM, hypertension, atherosclerotic cerebral infarction, coronary heart disease and others [39,40–42]. Recently, several miRNA target prediction databases, such as miRSNP [23], PolymiRTS [43], miRNASNP [44], have been established and may help us identify new functional polymorphisms in 3ˈUTRs. In the present study, our search of the miRSNP database identified another 3ˈUTR variant, i.e. +1451*C* > *T* (rs3025040), located in the miR-199a or miR-199b binding site that exhibited strong linkage (r^2^ = 1.0) with +936*C* > *T*. Further luciferase analysis revealed that the rs3025040 *T* allele significantly decreased luciferase activity in four cell lines, which supported our informatics-based prediction that +1451*C* > *T* may disrupt miRNA-mRNA interaction and result in lower VEGF expression levels. These results are consistent with many previous results that the rs3025039 *T* allele significantly decreased VEGF levels [35,36].

miR-199 is an important vertebrate specific miRNA family, which has been shown to be involved in a wide variety of cellular and developmental mechanisms such as various cancer development and progression, cardiomyocytes protection or skeletal formation [45]. Recently, a study had been reported that the expression of miR-199a and miR-199b was significantly lower in epileptic brain tissue than normal brain tissue [46]. Moreover, miR-199a and miR-199b had been found to regulate cell proliferation, survival and death by targeting HIF-1a [47]. HIF is an important transcription factor that regulates gene expression in the brain and other tissues in response to decrease in oxyone availability [48, 49]. Additionally, vascular endothelial cell is a crucial tissue to secrete VEGF after IS and induce the vascular regeneration. Chen T, et al. reported that miR-199b could modulate vascular cell fate through targeting the ligand Jagged1 and enhancing VEGF signaling [50]. These results suggested that miR-199a and miR-199b are important regulators to control the IS recovery.

Finally, although we did not evaluate any other possible mechanisms in the present study, such as AP4 for +936 *C* > *T*, no significant differences of relative luciferase activities were observed between the wil- and mut-types in the cells transfected with or without pcDNA3.1 empty vector groups, which suggested that other mechanisms may not play crucial roles in the expression differences observed between the different rs3025039 genotype.

In conclusion, in this case-control study, we observed that the +936 *C* > *T* polymorphism in the 3’UTR of *VEGFA* confers an increased risk of poor outcome at 90 days after IS onset. Additionally, based on a luciferase reporter assay, we have provided an initial description of the effect on the +1451*C* > *T* substitution on the binding of hsa-miR-199a and hsa-miR-199b that could result in decreased VEGF expression. The +936 *TT* genotype may be an important biomarker for the prediction of IS recovery in Chinese patients. Recently, some promoter polymorphisms in the region of *VEGFA*, such as -2578 *C*>*A*, -1154 *G*>*A* and -634 *C*>*G*, have been reported to affect the susceptibilities and outcomes of many common diseases [51–53]. Therefore, further studies are needed to investigate whether these polymorphisms and their combined effect influence VEGF expression and IS outcome. Finally, considering the small sample size in the present study, we did not evaluate genotype-environment interaction. Larger population-based studies with different ethnic groups are warranted to validate our findings.

## Supporting information

S1 Table The genotypes of +936 *C* > *T* and +1451 *C* > *T* of *VEGFA* gene in 36 ischemic stroke patients. (PDF)

S2 Table The effect of the variants on the the binding affinity of mir-199a and mir-199b within *VEGFA*-3’UTR. (PDF)

S1 Fig The effect of +936 *C* > *T* on the binding affinities between the *VEGFA* mRNA and miR-591. MFE: minimal free energy.
(TIF)

## Abbreviations

3ˈUTR: 3ˈ-untranslational regions
95%CI: 95% confidence intervals
BMI: body mass index
CT: computed tomography
DM: diabetes mellitus
GWAS: genome-wide association studies
IS: ischemic stroke
LAA: large artery atherosclerosis
LD: linkage disequilibrium
MFE: minimum free energy
miRNAs: microRNAs
MRI: magnetic resonance image
mRS: modified Rankin Scale
NIHSS: National Institutes of Health Stroke Severity Scale
OR: odds ratio
SAO: small artery occlusion
SNP: single nucleotide polymorphism
TOAST: Trial of Org 10172 in Acute Stroke Treatment
VEGF: Vascular endothelial growth factor
