# Supplementary material for: A functional variant in the 3ˈ-UTR of VEGF predicts the 90-day outcome of ischemic stroke in Chinese patients
Source: PLoS One. 2017 Feb 24;12(2):e0172709. doi: 10.1371/journal.pone.0172709 (PMC5325536; doi:10.1371/journal.pone.0172709)
Supplement: S1 Table — (PDF) [file pone.0172709.s001.pdf]

S1 Table. The genotypes of +936 C > T and +1451 C > T of *VEGFA* gene in 36 ischemic stroke patients

Sample ID	*VEGFA* genotype	
	rs3025039 (+936 C > T)	rs3025040 (+1451 C > T)
M2610	C/C	C/C
M2611	C/C	C/C
M2612	C/T	C/T
M2614	C/T	C/T
M2615	C/T	C/T
M2616	C/T	C/T
M2617	C/C	C/C
M2618	C/C	C/C
M2619	C/C	C/C
M2620	C/C	C/C
M2621	C/T	C/T
M2622	C/C	C/C
M2623	C/C	C/C
M2624	C/T	C/T
M2625	C/C	C/C
M2626	C/T	C/T
M2627	C/C	C/C
M2628	C/T	C/T
M2629	C/T	C/T
M3132	C/C	C/C
M3133	C/C	C/C
M3134	C/T	C/T
M3139	C/C	C/C
M3141	C/C	C/C
M3142	C/C	C/C
M3147	C/C	C/C
M3149	C/C	C/C
M3150	C/C	C/C
M3151	C/C	C/C
M3156	C/C	C/C
M3158	C/C	C/C
M3161	C/C	C/C
M3162	C/C	C/C
M3163	C/C	C/C
M3164	C/T	C/T
M3165	C/C	C/C
