# Supplementary material for: A functional variant in the 3ˈ-UTR of VEGF predicts the 90-day outcome of ischemic stroke in Chinese patients
Source: PLoS One. 2017 Feb 24;12(2):e0172709. doi: 10.1371/journal.pone.0172709 (PMC5325536; doi:10.1371/journal.pone.0172709)
Supplement: S2 Table — (PDF) [file pone.0172709.s002.pdf]

S2 Table. The effect of the variants on the the binding affinity of mir-199a and mir-199b within *VEGFA*-3'UTR.

Groups	Cell types	N	Related luciferase activity		Inhibit rate (%)	*P* value*	Combined *P* value#
			C	T			
pcDNA3.1	A549	3	1.000±0.016	1.000±0.015	---	---	---
	16HBE	3	1.000±0.032	1.000±0.035	---	---	
	293T	3	1.000±0.020	1.000±0.011	---	---	
	ECV304	3	1.000±0.017	1.000±0.028	---	---	
mir-199a	A549	3	0.894±0.033	0.747±0.063	15.9%	0.083	<0.001
	16HBE	3	0.787±0.032	0.565±0.061	28.1%	0.062	
	293T	3	0.675±0.034	0.518±0.060	23.3%	0.002	
	ECV304	3	0.554±0.033	0.451±0.023	23.0%	0.107	
mir-199b	A549	3	0.909±0.014	0.799±0.008	12.3%	<0.001	<0.001
	16HBE	3	0.746±0.058	0.664±0.069	11.0%	0.153	
	293T	3	0.883±0.032	0.749±0.070	15.1%	0.145	
	ECV304	3	0.679±0.062	0.542±0.053	19.3%	0.130	

***** pairwise t test # The comparison between the C and T alleles in all four transfected cell lines using pairwise t test.
