# Supplementary figures and images for: A functional variant in the 3ˈ-UTR of VEGF predicts the 90-day outcome of ischemic stroke in Chinese patients
Source: PLoS One. 2017 Feb 24;12(2):e0172709. doi: 10.1371/journal.pone.0172709 (PMC5325536; doi:10.1371/journal.pone.0172709)

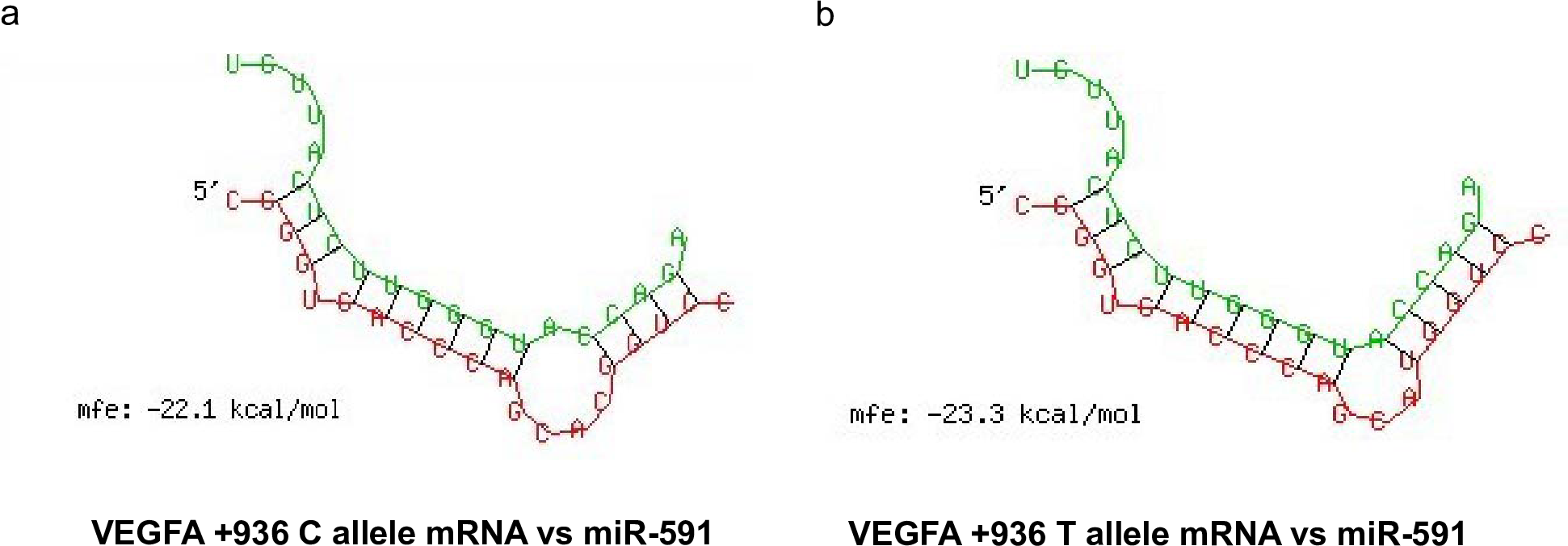

Supplement: S1 Fig — MFE: minimal free energy. (TIF) [file pone.0172709.s003.tif]
